# Survey on awareness of artificial intelligence in pediatric dentistry

**DOI:** 10.6026/973206300211647

**Published:** 2025-06-30

**Authors:** Shilpa S. Naik, Jasmin Winner, Ishani Ratnaparkhi, Shirin Chavan, Mayuri Gorule, Ruchi Chaudhary

**Affiliations:** 1 Department of Pediatric Dentistry, D.Y. Patil Deemed to be University, School of Dentistry, Navi Mumbai, Maharashtra, India

**Keywords:** Artificial intelligence, pediatric dentistry, diagnostic accuracy, treatment planning

## Abstract

Artificial intelligence (AI) is transforming pediatric dentistry by improving diagnostic accuracy, treatment planning and patient care. Therefore, it is of interest to assess the knowledge, awareness and attitudes of pediatric dental professionals in India toward AI integration. While the majority recognized the benefits of AI, significant knowledge gaps and barriers such as cost, ethical concerns and lack of training were identified. A statistically significant association was found between awareness levels and years of experience as well as practice settings. These findings underscore the need for structured educational programs to support AI adoption in pediatric dentistry.

## Background:

Artificial intelligence (AI) refers to computer systems capable of performing tasks that typically require human intelligence, including data analysis, pattern recognition and decision-making [1]. In healthcare, AI has shown significant promise in improving diagnostic precision, streamlining workflows and enhancing patient outcomes [2]. Dentistry is gradually incorporating AI-driven technologies such as diagnostic imaging, treatment planning tools and predictive analytics, which assist clinicians in making data-informed decisions [3]. In pediatric dentistry, where behavior management, early diagnosis and personalized care are essential, AI offers notable advantages [4]. Tools such as virtual reality (VR), AI-assisted teledentistry and predictive models can improve diagnostic efficiency, reduce anxiety in children and expand access to care in underserved areas [5, 6]. However, current literature suggests limited awareness and preparedness among pediatric dental professionals regarding AI integration into clinical workflows [7]. Therefore, it is of interest to report the level of knowledge, awareness and readiness for AI adoption among pediatric dentists through a structured survey.

## Materials and Methods:

## Study design:

This study was conducted as an observational, cross-sectional survey. It targeted practicing pediatric dentists across various dental settings, including private clinics, academic institutions and hospital-based practices located in India.

## Participant selection and sample size:

Inclusion criteria were established to include only pediatric dentists with recognized qualifications and active clinical or academic practice. Exclusion criteria encompassed general dentists, dentists from other specialties and postgraduate students enrolled in pediatric dentistry programs at the time of the study. The sample size was determined based on the estimated pediatric dentist population and expected response rate. A standard formula for cross-sectional surveys, factoring in a 95% confidence level and an assumed awareness proportion (p), was utilized. A final sample of 300 potential participants was identified to account for possible non-responses and incomplete questionnaires, consistent with recommendations in similar cross-sectional survey studies.

## Questionnaire development and validation:

The survey questionnaire was developed after reviewing existing literature on AI applications in dentistry and previous survey-based research [8]. A draft version was then evaluated by six senior pediatric dentists for content clarity and relevance. Their feedback resulted in minor modifications to question wording and structure. Pilot testing was conducted with a small group of 20 pediatric dentists, who did not form part of the final sample, to ensure comprehension and test survey flow. The final questionnaire featured close-ended items with Yes/No responses, multiple-choice questions and Likert-scale ratings where appropriate, focusing on assessing participants' knowledge, attitudes and perceived barriers regarding AI in pediatric dentistry.

## Data collection procedures:

Participants were invited via email, containing an explanation of the study's purpose, a consent statement and a secure link to the online questionnaire (hosted on Google Forms). Data collection occurred over a one-month period. To maximize the response rate, reminder emails were sent biweekly and participants were assured of anonymity and confidentiality. All responses were automatically recorded in a secure spreadsheet linked to the questionnaire platform.

## Ethical considerations:

Approval for the study was obtained from the Institutional Ethical Board, under the reference number IREB/2024/PEDO/24. Participation was voluntary, with all respondents providing informed consent before accessing the questionnaire. No personally identifiable information was collected and all data were stored in password-protected systems with restricted access to the principal investigators.

## Statistical analysis:

Upon completion of data collection, the responses were exported to IBM SPSS (version 17.0) for analysis. Descriptive statistics (frequency, percentage) were used to summarize participant demographics and question responses. The chi-square test was employed to evaluate any associations between demographic variables (*e.g.*, years of experience) and questionnaire responses. A p-value < 0.05 was considered statistically significant.

## Results and Discussion:

A total of 400 pediatric dentists responded to the online questionnaire, giving a response rate of 66.67%. Of these, 380 responses were complete and eligible for analysis. The sample represented a range of age groups and professional settings (Table 1 - see PDF). The responses of the participants to each question are collectively summarized in Table 2 (see PDF).

The survey revealed that 65% of participants were aware of AI applications in pediatric dentistry, while 22.5% were unaware and 12.5% were uncertain. AI awareness was highest among dentists working in academic settings (72%), which may reflect greater exposure to research and continuing education. In contrast, private practitioners reported lower familiarity (60%), which may indicate limited access to AI-focused learning modules. This difference underscores the influence of professional environment on technology awareness and supports findings from Tandon and Rajawat (2020), who emphasized the role of institutional support in AI literacy [9]. When asked about the clinical utility of AI, 70% of participants agreed that AI-assisted care would be beneficial in pediatric dentistry. Specifically, 62.5% felt AI could help predict dental issues in children using patient data, risk factors and behavioral trends. This is consistent with studies such as Shan *et al.* (2021) that highlight the ability of AI to detect caries risk and intercept malocclusion early [10]. Such predictive tools can shift pediatric care toward prevention-based strategies and reduce the need for invasive interventions. A notable 67.5% of respondents acknowledged the potential of AI-driven tools like VR to reduce anxiety during dental procedures. VR has been used to create immersive environments that distract pediatric patients during treatment [11]. These findings confirm the interest in non-pharmacological behavior management approaches and demonstrate the relevance of AI beyond diagnostics. Approximately 73.75% believed that AI could improve access to pediatric dental care in underserved regions through technologies like teledentistry. The acceptance of AI in outreach settings is encouraging, especially in a country like India where geographic disparities in specialist availability persist. Recent work by Perez *et al.* (2025) similarly found that AI-powered teledentistry could bridge rural access gaps [12]. Further, 56.25% agreed that AI could facilitate oral health monitoring in children with special healthcare needs via wearables and remote technologies. This reflects growing awareness of how AI can support continuity of care for vulnerable pediatric populations, aligning with findings by Vishwanathaiah *et al.* (2023) on AI-enabled remote monitoring systems [13]. However, despite the enthusiasm, 71.25% of participants cited the high cost of AI equipment and slow return on investment as major adoption barriers. This concern was particularly pronounced among private practitioners, likely due to limited institutional funding. Davenport and Kalakota (2019) also noted that financial constraints remain one of the primary impediments to AI adoption in clinical practice [14]. Ethical and data privacy concerns were raised by 45% of respondents, pointing to apprehensions around the handling of sensitive pediatric health data, a theme echoed in an earlier work [16]. Only 60% of respondents said they would be willing to try AI-powered teledentistry tools, while 20% remained unsure. This suggests that while the concept is attractive, uncertainty about reliability, patient acceptance and integration with existing workflows still persists [16]. Additionally, 52.5% felt AI could help children maintain good oral hygiene through gamified apps and digital coaching. While this is a promising trend, 25% were unsure about its practical effectiveness, perhaps reflecting limited exposure or skepticism regarding long-term behavioral impact. Statistical analysis revealed a significant association between years of clinical experience and AI awareness (p < 0.05), with more experienced clinicians exhibiting slightly less familiarity. This finding may stem from generational differences in technological adoption and training exposure. A separate chi-square analysis indicated that hospital-based dentists were more open to AI-powered imaging tools (p = 0.04), likely due to regular interaction with digital radiographic systems. Meanwhile, cost-related hesitations were significantly higher among private practitioners (p = 0.02), reinforcing economic barriers in solo or small group practices. Supplementary comments by respondents emphasized a lack of formal training as a critical barrier to AI integration. Many felt that AI is not adequately addressed in current pediatric dentistry curricula. Concerns were also raised about AI potentially undermining human empathy, particularly in behavior management. These insights reinforce the importance of structured AI education, ethical standards and maintaining a humanistic approach in pediatric dental care [13]. This study advances existing literature by providing context-specific data on Indian pediatric dentists' knowledge, attitudes and readiness toward AI. While earlier studies have explored AI in general dentistry or radiology, few have focused specifically on pediatric dentistry. Our findings demonstrate a positive perception of potential of AI, tempered by realistic concerns about cost, ethics and implementation readiness. As AI technologies become more accessible and user-friendly, continued education and supportive policy will be critical in facilitating their responsible adoption in pediatric practice.

## Conclusion:

Pediatric dentists showed moderate to high awareness of AI, with strong interest in its clinical applications. Key barriers such as cost, privacy concerns and limited training need urgent attention. Structured education and clear implementation guidelines are essential for responsible AI integration in pediatric dentistry.
